# Social capital and maternal and child health services uptake in low- and middle-income countries: mixed methods systematic review

**DOI:** 10.1186/s12913-021-07129-1

**Published:** 2021-10-22

**Authors:** Endalkachew Worku Mengesha, Getu Degu Alene, Desalegne Amare, Yibeltal Assefa, Gizachew A Tessema

**Affiliations:** 1 Department of Reproductive Health and Population Studies, School of Public Health, College of Medicine and Health Sciences, Bahir Dar University, Bahir Dar, Ethiopia; 2 Department of Epidemiology and Biostatistics, School of Public Health, College of Medicine and Health Sciences, Bahir Dar University, Bahir Dar, Ethiopia; 3 School of Health Sciences, College of Medicine and Health Sciences, Bahir Dar University, Bahir Dar, Ethiopia; 4 School of Public Health, the University of Queensland, Brisbane, Australia; 5 Curtin School of Population Health, Curtin University, Perth, WA Australia

**Keywords:** Maternal and child health services, Social capital, Social network, LMICs

## Abstract

**Background:**

Social capital has become an important concept in the field of public health, and is associated with improved health services uptake. This study aimed to systematically review the available literature on the role of social capital on the utilization of maternal and child health services in low- and middle-income countries (LMICs).

**Methods:**

Mixed-methods research review and synthesis using three databases PubMed, Scopus, and Science Direct for peer-reviewed literature and Google Scholar and Google search engines for gray literature were performed. Both quantitative and qualitative studies conducted in LMICs, published in English and in grey literature were considered. Prior to inclusion in the review methodological quality was assessed using a standardized critical appraisal instrument.

**Results:**

A total of 1,545 studies were identified, of which 13 records were included after exclusions of studies due to duplicates, reading titles, abstracts, and full-text reviews. Of these eligible studies, six studies were included for quantitative synthesis, and seven were included for qualitative synthesis. Of the six quantitative studies, five of them addressed the association between social capital and health facility delivery. Women who lived in communities with higher membership in groups that helps to form intergroup bridging ties had higher odds of using antenatal care services. Synthesized qualitative findings revealed that women received some form of emotional, informational, and instrumental support from their network members. Receiving health information from trusted people and socio-cultural factors influenced the use of maternal and child health services.

**Conclusions:**

Social capital has a great contribution to improve maternal and child health services. Countries aiming at improving maternal and child health services can be benefited from adapting existing context-specific social networks in the community. This review identified limited available evidence examining the role of social capital on maternal and child health services uptake and future studies may be required for an in-depth understanding of how social capital could improve maternal and child health services.

**Systematic review registration:**

PROSPERO CRD42021226923.

**Supplementary Information:**

The online version contains supplementary material available at 10.1186/s12913-021-07129-1.

## Background

Maternal and child mortality is declining in the last two decades but remains relatively high in the low- and middle-income countries (LMICs). About 86 % of the global maternal deaths occurred in two regions, sub-Saharan Africa (SSA) (accounted for 66 %), and southern Asia (accounted nearly 20 %) [1–3]. Although global neonatal mortality rate has declined by half between 1990 and 2017, over 2.5 million children are still dying in the first month of life [4]. Sustainable Developmental Goals (SDGs) have target of less than 70 maternal deaths per 100,000 live births and a reduction in under-5 mortality to 25 per 1000 live births by 2030. The ambition of these SDGs targets can be achieved by improving maternal and child health (MCH) services uptake, especially in the high-burden regions of south Asia and SSA [4].

Provisions of MCH services are essential for the early detection of mothers and infants at high risk of morbidity and mortality [5, 6]. Maternal and child health services are series of interlinked healthcare services provided during pregnancy, childbirth, and postpartum periods. These services has been advocated for improving MCH as each stage builds on the success of the previous stage [7]. For example, a systematic review conducted in east African countries showed that women who received antenatal care (ANC) services are more likely to attend postnatal care (PNC) services than those who did not received ANC [8]. Although there have been improvements in MCH services coverage globally, overall MCH indicators remained low with significant disparities between the lowest and highest wealth quintiles [9, 10]. Studies in LMICs showed that high maternal and child mortality was highly related to low level of ANC visits, health facility delivery, immunization, decision making capacity and social capital scores [8, 11–17]. Social capital can play a role in improving MCH services uptake and it has been positively related to physical and mental health of members in the social networks [18, 19].

Social capital has multiple definitions and concepts in the field of economics, sociology, political science, and other disciplines [20]. Recently, it has become an important concept in field of public health [21] and is defined as social relations that may provide individuals and groups with access to resources and supports in their community networks. It may include different forms such as exchange of favors, maintenance of group norms, trust towards individuals or groups, and supports offered to members of social groups [22]. A number of social capital theories were grounded so far and growing from individual and family property to features of communities and nations [23–25]. The theory of social capital can be explained in structural and cognitive forms. In the structural form, it focuses on the externally observable aspects of social organizations and refers to the intensity of an individual’s participation in community networks measured in objective terms [26]. The cognitive form involves subjective aspects such as norms, values, attitudes and perceptions of an individual’s social relationship and can be measured subjectively. Structural and cognitive forms of social capital are not mutually exclusive and characterized in terms of social relations as what people ‘do’ and what people ‘feel’, respectively [18, 27, 28].

Evidence on the role of social capital and MCH has grown in recent years; however, most of these studies were conducted in high-income countries, such as the Netherlands [29, 30], USA [31, 32], UK [33] and Spain [34]. However, there are some studies from LMICs, where socio-economic inequality is higher, reported that social capital has a stronger relationship and a greater effect on health [35, 36]. Our preliminary cursory searches identified additional studies conducted in India [27, 37], Tanzania [38] and Cameroon [39]. In other African countries such as Ethiopia, there is high levels of group membership, high participation in citizenship activities and high levels of cognitive social capital [40]. However, its benefit to improve women and families access to healthcare is not well studied. For example, women and family members who are involved in social networks including ‘*Debo*’ and *‘Iqqub’* in Ethiopia could offer great opportunities for them to receive important health information. These networks also usually established for economic and social supports to the members and families. *‘Debo’* is working together informal grouping in the community to help each other for farming, house building and construction in Ethiopia community. *‘Iqqub’* is a common financial assistance association where families, friends or other groups contribute some money together and share the money in rounds for each of the contributors in a specified time period [41].

To date, there was no systematic or narrative review that systematically synthesizes the available literature focusing on the role of social capital on MCH services use in LMICs. Therefore, this review aimed to synthesize the available quantitative and qualitative literature on the role of social capital on MCH services uptake in LMICs. The findings of the study will inform policy and decision makers to improve MCH services uptake in LMICs.

## Methods

This study followed mixed methods systematic review (MMSR) approach developed by the Joanna Briggs Institute (JBI) for mixed evidence synthesis. The MMSR is an emerging systematic review approach that can bring together the findings of quantitative and qualitative evidence to provide comprehensive evidence to enhance the findings applicability for decision-makers [42]. The protocol of this review was registered with the PROSPERO database (registration number: CRD42021226923). The review also followed Preferred Reporting Items for Systematic Reviews and Meta-Analyses (PRISMA) guidelines (Supplementary file 1) [43].

### Inclusion criteria

In this systematic review, the inclusion criteria were:

- Types of studies: both quantitative and qualitative studies.
- Publication status: both published and unpublished/grey literature.
- Language: articles and reports written in English.
- Population: studies reporting on women and children were considered.
- Intervention: social capital was the intervention for our study.
- Context: studies that were conducted in one or more of the LMICs based on the World Bank criteria.
- Outcome reported: studies that reported on the association of social capital and MCH services were considered. In this review, MCH services comprised ANC, health facility delivery and PNC use. The phenomenon of interest for the qualitative component included experiences/views/perceptions of women on the role of social capital on MCH services uptake.

### Search and search strategy

We used PubMed, Scopus and Science Direct databases to search peer-reviewed articles. In addition, Google and Google Scholar search engines were used to identify grey literature and government reports. A comprehensive search strategy was initially developed by EWM and reviewed by GAT and YA. Medical subject headings (MeSH), text words and key words in the title or abstract were considered when developing search strategy. Our search strategy combined terms related to the three domains: In the first category, the term related to ‘social capital’ such as ‘social support’, ‘social trust’, ‘social network’, ‘community network’, OR ‘social cohesion’; in the second category, ‘maternal and child health services’ terms that comprised ‘antenatal care’, ‘health facility delivery’ OR ‘postnatal care’; in the third category, country terms such as ‘LMICs’ regions were included. Lists of LMICs were found from recent World Bank classification [44]. All synonym keywords and subject headings were combined with the “OR” Boolean operator. Finally, we combined all the three categories using the Boolean operator “AND” to run in the databases (Supplementary file 2). When we search in Google Scholar and Google search engines, the first 100 hits were included. Moreover, we also reviewed websites of international organizations such as the World Health Organization and the World Bank for unpublished reports.

### Study selection

**Fig. 1:**
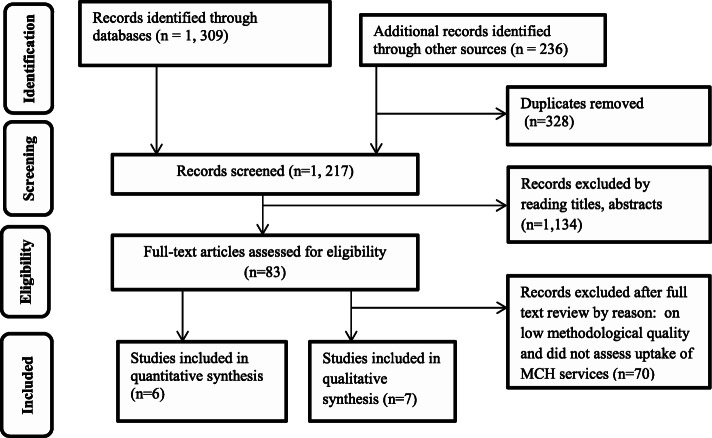
PRISMA flow diagram summarizing selection of studies included in the mixed evidence systematic review

Once literature searching was completed, studies were screened for eligibility by EWM and DA independently by considering the inclusion criteria after retrieved records were exported into EndNote. Any disagreements on article exclusion or inclusion were resolved by consensus. A total of 1,545 studies were identified by searching databases and other search engines to synthesize evidence about the role of social capital on uptake of MCH services in LMICs, of which, 328 records were removed due to duplication. Then 13 records were selected after reading titles, abstracts, and full texts. The reasons for exclusions after full text review included low methodological quality, and did not assess uptake of MCH services. (Fig. 1)

### Data extraction and management

Data were extracted from articles or reports using an excel sheet. The main information collected from each study contain last name of author(s), year of publication, study methods (study setting, study participants, study design, the year of data collection, sample size and data analysis), key findings and limitation acknowledged by the author(s) of the study.

### Data synthesis

For the quantitative studies, we performed textual narrative synthesis after tabulating individual studies. Due to lack of uniformity on the definitions and measurement of social capital, we were not able to conduct meta-analysis. Descriptive characteristics, key findings, and limitations of individual studies were presented in tables. For the qualitative evidence, findings were synthesized using meta-aggregation approach. Initially, views/experiences of mothers on social capital were synthesized. Then pooled findings were grouped into categories defined by their similarity of meaning and then combined into one or more synthesized finding(s) that captured their meaning.

### Methodological quality assessment

Both quantitative and qualitative studies selected for retrieval were assessed by two independent reviewers (EWM and DA) for methodological validity prior to inclusion in the review using standardized critical appraisal instrument. The criteria were adapted from the JBI Critical Appraisal Checklist and classified as high quality: meets ≥ 7 criteria, moderate quality: meets ≥ 4 criteria, and low quality: meets < 4 criteria [45].

Any disagreements that arise between the reviewers were resolved through discussion. The results of critical appraisal were reported in narrative form and in a table. Assessment of methodological quality, or critical appraisal, was conducted to establish the internal validity and risk of bias of studies that meet the review inclusion criteria. We checked the appropriateness of study methodology for addressing the research question and interpretation of findings in a way that is appropriate to the methodology. (Supplementary file 3)

## Results

### Characteristics of the studies

A total of 13 studies were included for this systematic review. Of these eligible studies, six studies [27, 37–39, 46, 47] were included for quantitative synthesis and seven [48–52] of them for qualitative synthesis. Based on the income distribution of the studies, eleven studies came from lower middle-income countries, and the rest two belongs to low income countries. In relation to geographical distribution, seven countries came from south Asian countries, and six studies appeared from SSA countries. Most of the included quantitative studies (*n* = 5) investigated the association between social capital and health facility delivery. Three studies reported the relationship between social capital and ANC service. (Tables 1 and 2)

**Table 1:** Quantitative studies included in a systematic review of the role of social capital on maternal and child health services uptake in LMICs

First author/publication year	Aim(s) and study Design	Country and year of study	Study participants and sample size	Data collection method(s)	Data analysis	Outcome measurement	Estimate for social capital	Limitation(s) of the study identified by the author(s)
Singh et al., 2014 [37]	Aim: to examine factors associated with maternal healthcare utilization in nine high focus states Design: Secondary analysis of cross sectional study	India, 2007-08	125,721 ever-married women aged 15–49	A set of structured questionnaires namely, household, ever married woman, unmarried woman, village questionnaires and health facility survey	Multilevel analyses	≥ 4 ANC visits	• Individual/household level Social group: Scheduled Tribes; AOR = 0.83 (0.80–0.87) Scheduled Castes; AOR = 0.90 (0.87–0.92)	• Recall bias since information was collected retrospectively, women may overlook or may not accurately recall the number or timing of prenatal care, location, and attendant of birth, or PNC during interview • Not all predictors of maternal healthcare services use were included • limitation in considering measures of quality of healthcare services such as waiting time, staff attitudes and behavior
						Health facility delivery	• Individual/household level Social group: Scheduled Tribes; AOR = 0.83 (0.80–0.86) Scheduled Castes; AOR = 0.91 (0.89–0.94)	
						PNC within 2 days after delivery	• Individual/household level Social group: Scheduled Tribes; AOR = 0.91 (0.88–0.95)	
Story et al., 2014 [27]	Aim: to examine the association between social capital and the utilization of antenatal care, professional delivery care, and childhood immunizations Design: Cross sectional study	India, 2005	10,739 women who recently gave birth and 7,403 children between one and five years of age in 2,293 communities and 22 state-groups	Household interviews were conducted with ever-married women aged 15–45	Multilevel logistic regression Exploratory factor analysis	≥ 4 ANC visits	• Individual/household level: Social networks (AOR = 1.10) • Community level: Intergroup bridging ties (AOR = 1.22) Intragroup bonding tie (AOR = 0.83) Collective efficacy (AOR = 0.90)	• The study was not designed to infer a causal association due to the retrospective, cross-sectional nature of the data. • No way to differentiate between male and female participation in the social capital questions • Measurement of each component of social capital was limited by the questions that were used in the survey
						Health facility delivery	• Community level: Intragroup bonding tie (AOR = 1.13) Social networks (AOR = 1.16) Social cohesion (AOR = 0.90) Collective efficacy (AOR = 1.09)	
Semali et al., 2015 [38]	Aim: to determine the role of social capital in facilitating health facility delivery Design: Community based cross sectional study	Tanzania, 2015	744 mothers with children aged less than five years	Validated World Bank’s social capital assessment tool was used [68]. Questionnaire administered in face-to face interviews.	Multilevel analysis and Principal Component Analysis	Health facility delivery	Social capital quintiles: Lowest; AOR = 2.9 (1.4–6.1) Moderate, AOR = 5.5 (2.3–13.3) High; AOR = 4.7 (1.9–11.6) Highest; AOR = 5.6 (2.4–13.4)	Mothers who survived the birth process and hence introduced a bias which might have overestimated the rate of facility deliveries
Saha et al., 2013 [46]	Aim: to analyze the impact of self-help groups on maternal health service uptake at national level Design: secondary analysis of cross sectional study	India, 2013	643,944 ever married women (15–49 years)	Data was collected through self-reported information from respondents	Forward stepwise logistic regression model	Health facility delivery	Presence of self-help group: AOR = 1.19 (1.13–1.24)	• Information on women’s actual participation in self-help group activities was not included • Analysis done at the aggregate country level. This masks variations in the spread and intensity of self-help group activity • The availability of credit and the duration of association did not addressed • An explicit definition of self-help group was not stated • The design and nature of the study were not able to draw conclusions about causality
Mohammed et al., 2019 [47]	Aim: to examine the association between male partners’ involvement in maternal health care on utilization of maternal health care services Design: community-based cross-sectional study	Ethiopia, 2014	210 male/female couples with a baby less than 6 months old	Two structured questionnaires were used to collect the data from men and women	Multivariate logistic regression models	At least one ANC visit	Overall male partners’ involvement (MPI) scale score: AOR = 1.61 (1.05–2.45)	Self-report might introduced social desirability bias
						Health facility delivery	Overall MPI scale score: AOR = 1.22 (1.01–1.48)	
McTavish et al., 2015 [39]	Aim: to examine the importance of social networks and social capital in maternal health care use Design: cross-sectional study	Cameroon, 2009	110 women between 18–45 years old who had given birth at any time in the five years prior	Interviews were conducted	Poisson regression and inductive content analysis	Number of maternal health care visits	Network resources Incidence rate ratios (IRR) = 1.13 (1.02–1.26)	Results may not be generalizable to other populations due to convenient sampling techniques

*ANC:* Antenatal Care, *AOR*: Adjusted Odds Ratio, *IRR:* Incidence Rate Ratios, *MPI*: Male Partners’ Involvement, *PNC*: Postnatal Care

**Table 2:** Qualitative studies included in the review of the role of social capital on maternal and child health services uptake in LMICs

First author/publication year	Aim(s) and study Design	Country and year of study	Study participants and sample size	Data collection method(s) and analysis	Social capital measures	Description of social capital findings	Limitation(s) of the study identified by the author(s)
Cofie et al., 2018 [51]	Aim: to examine the social network dynamics of all members of women’s social networks during pregnancy and childbirth Design: Phenomenology	Ghana, 2015	• Mothers (*n* = 40) • Husbands (*n* = 20), and • 4 focus group interviews with mothers-in-law	• In-depth interviews (IDIs) • Focus group discussions(FGDs) • Data were analyzed using narrative summaries and thematic coding	Social support and network: Network proximity Frequency of contact Nature of relationships	Social networks contribute in important ways to women’s use of facility-based pregnancy and delivery care	Translation errors, Recall bias, Response bias, Social desirability bias
Mochache et al., 2020 [52]	Aim: to explore how individual and community-wide factors influenced uptake and utilization of maternal health services Design: Phenomenology	Kenya, 2015	• Female (pregnant and postpartum) as well as male adult community members • 5 FGDs (*N* = 47 ) • 15 IDIs(*N* = 15)	• FGDs stratified by age and gender; 3 among men and 2 among women, • IDIs • A thematic content analytic approach was used	Socio-cultural norms, religious norms and gender stereotypes	Religious and socio-cultural norms as well as gender stereotypes influenced utilization of maternal health services	No limitation information was provided
Papp et al., 2013 [49]	Aim: to identify the processes and psycho-social pathways through which social accountability can contribute to improvement of maternal health Design: case study	India, 2013	• Interviews with 4 health providers, • 3 policy-makers and government officials, • 4 media representatives, • 2 representatives from partner, • 2 national, 4 state and district Central Statistical Agency staff, • 1 Accredited social health activist	Interviews and focus groups	Critical consciousness, social capital and ‘receptive social spaces’ to outline a social-psychological account of the pathways between Social accountability and service effectiveness	Three processes that underpin social accountability: (1) generating demand, (2) leveraging intermediaries and (3) sensitizing leaders and health providers to the needs of women.	Focused on the processes and psycho-social pathways underpinning the public hearings
Raman et al., 2014 [50]	Aim: to explore the wide-ranging sources of support that the maternal–infant dyad need or expect throughout the perinatal period Design: qualitative interviews and ethnographic approach	India, 2008-10	• 36 mothers from different socio-cultural and socio-economic backgrounds who had given birth within the past two years in a tertiary hospital • 13 participants in group one (low education), • 11 in group two (medium education) and • 12 in group 3 (high education).	• IDIs • Thematic analysis of transcribed interviews • Ethnographic field notes was carried out	• Female networks • Extended family support • Own mother emotional support and advice	4 themes emerged: • Importance of women’s own mothers • My place • Female support network • Role of husband • The ambivalent role of the family	No limitation information was provided
Mamo et al., 2019 [48]	Aim: to explore the actual roles, responsibilities, and contribution of different community individuals or groups in promoting ANC, childbirth and early postnatal cares. Design: case study	Ethiopia, 2016	HEWs, religious leaders, Women Developmental Army leaders, Male Developmental Army leaders and married male and female community members	12 FGDs and 24 semi-structured IDIs	Social support Provision of continuous support Work as a community-health care system linkages	Offering social support (practical help with routine activities, resources and material goods, emotional support and assurance, nutritional support, and accompaniment)	• Unable to explore information from zonal health officers • Specific distance from a woman’s residence to a health facility were not explicitly accounted in this study • Social desirability bias
Simkhada et al., 2010 [53]	Aim: to explore the mother-in law’s role in (a) her daughter-in-law’s ANC uptake; and (b) the decision-making process about using ANC services in Nepal. Design: Exploratory qualitative study	Nepal, 2006	• 30 purposively selected antenatal or postnatal mothers (half users, half non-users of ANC), • 10 husbands and • 10 mothers-in-law in two different (urban and rural) communities	IDIs	Communication and relationships between mothers-in-law and daughters-in-law	• Use of ANC is strongly influenced by mothers-in-law’s roles and attitudes • Mothers-in-law appeared to have less influence on ANC uptake if they did not live in the same household as their daughters-in-law	• It was not feasible to include literate mothers-in-law
Sapkota et al., 2012 [54]	Aim: to explore husbands’ experiences of supporting their wives during childbirth Design: Exploratory qualitative study	Nepal, 2009	• 12 fathers who had supported their wives during childbirth	IDIs	Husbands helped to be present at the birth.	Despite the unpleasant emotions, a majority of the husbands felt that they were able to support their wives to some extent.	• Husbands in this study are from an urban setting, where people’s educational qualifications and their access to maternity health services are high

*ANC:* Antenatal Care, *FGDs:* Focus Group Discussions, *IDIs:* In-Depth Interviews

### Quality assessment of included studies

Of the six quantitative studies, five [27, 37, 39, 46, 47] were rated as moderate quality and one [38] was rated high quality. Four out of six studies undertook systematic random sampling in selecting their study participants. The samples taken for the study were representative and outcomes were measured using reliable methods. All of the six studies assessed their outcome using objective measures through proxy questions; controlled confounding factors using multivariate and multivariable regression analysis and did not describe those participants who withdraw or refused to participant in the study.

Regarding the qualitative synthesis, six out of the seven primary qualitative studies were assessed as high quality [48, 50–54]. The studies lacked description of the congruency between the philosophical perspectives and research methodology used. Moreover, the failure to describe the researchers’ perspectives may influence the analysis and interpretation of findings.

### Measurement of social capital

Studies included in this review reflected multiple dimensions of social capital. While some studies assessed both structural and cognitive social capital [37, 46, 47], other studies also examined bonding and bridging dimensions [27, 38]. Related to measurement tools for social capital, studies used different types of measurement tools ranging from using individual questions for selected dimensions of social capital to composite tools that measured each dimension of social capital using several questions. The variations among tools in its content may indicate that instruments for measuring social capital are at the developmental stage. Exploratory factor analysis (EFA) was used to develop and validate a tool for measuring social capital and investigate the influence of socio-contextual variables [27, 55–59]. In addition, confirmative factor analysis (CFA) indicated the reliability and validity of social capital scales [56, 60]. Furthermore, regression analyses including multiple hierarchical linear regression [61], multilevel models [27, 37, 62], multivariate regression [63, 64], multiple linear regression and logistic regressions [46, 65, 66] were used to examine the relationship between social capital and the outcome variables. For qualitative studies, data were analyzed using narrative summaries and thematic analysis approach [48, 50–52].

### Role of social capital on uptake of maternal and child health services

Of six reviewed quantitative studies, five of them addressed the relationship between social capital and health facility delivery. However, the six study included in our study examined the association between social networks and maternal health services during pregnancy. A study from rural India [46] showed that women from villages with a social capital in term of a self-help group were more likely to give birth in a health facility. In contrast, other study from India [67] reported that although the presence of self-help groups in the community, there was no evidence suggesting social networks would improve health facility delivery. These discrepancies could be women used self-help group for financial purpose only and they did not access health care messages during their meetings. (Table 1)

Three of the six studies [27, 37, 47] focused on the association between social capital and ANC uptake. Women who lived in communities with higher membership in groups that help form intergroup bridging ties had higher odds of ANC use than women who lived in communities with higher intragroup bonding ties and collective efficacy [27]. Furthermore, male partners’ involvement in maternal health care during pregnancy has improved maternal health care services access and uptake. The odds of having at least one ANC was higher in women whose male partners’ involvement scores were higher than those women whose partner’s involvement scores were lower [47].

### Experience of women on social capital

A total of seven qualitative studies were included in this systematic review. These studies followed phenomenology [51, 52], case study [48, 49], ethnography [50] and exploratory [53, 54] qualitative study designs. The synthesized qualitative findings of this study revealed that social support groups, receiving health information from trusted people and sociocultural factors played a significant role on the uptake of MCH services. Across network support groups, most women indicated that network members provided emotional, informational, and instrumental supports. Health extension workers (HEWs), women development army (WDA), Male development army (MDA) and religious leaders were also participated on community mobilization activities, provision of continuous support and promotion of MCH services [48–54]. For example, a study in Ethiopia reflected that religious leaders were crucial in providing emotional and spiritual support to their followers by committing themselves in prayer activities. This was illustrated when a female FGD participant said : *“In the Orthodox Church, while the religious leaders pray, they also pray for pregnant women and for women in postpartum period…to live in agreements as our governments ordered us, the church also ordered us too and made pray for us, we also pray at home”* (Female FGD participant) [48]. It was also shown that religious leaders were found to be influential in many aspects of community wellbeing including by the promotion of safe motherhood. *“…Now we are advising pregnant women to give birth in health facility. We also advise them to attend health checkup during pregnancy and go directly to health facility when labor starts. We are advising people to stop harmful practice like massaging the belly of pregnant women”* (Muslim religious leader) [52].

Other evidence showed that some women had numerous sources of support that include their own mothers, female relatives and friends. Female friendships were particularly important in those who lived in nuclear households or those who were far away from their own families [50]. Husbands had helped to boost their wife’s confidence and reduce anxieties during the childbirth. Regarding the community social networks, the benefit may be direct, through the young person’s own networks, or indirect, through the networks of their parent(s). Network support for women’s pregnancy-related care affects their place of childbirth. Women who had positive experiences of having health facility delivery previously and who lived in close proximity with the pregnant women tend to encourage women to consider birth at health facility. Those husbands lived in the same house further confirmed the emotional supportive roles of these network members [51]. Studies conducted in Ethiopia [48], India [49, 50], Ghana [51] and Kenya [52] demonstrated that women received some form of emotional, informational and instrumental support from their network members during pregnancy and to use health facility delivery [51].

Receiving health information from trusted people and socio-cultural factors influenced use of MCH services. A study conducted in Ethiopia [48] and Kenya [52] showed that religious and socio-cultural norms as well as gender stereotypes influenced uptake of MCH services. In Nepal, mothers-in-law played ambivalent role in the uptake of ANC. They have a positive influence when encouraging women to seek ANC, but more often a negative role in discouraging women from accessing ANC. Mothers-in-law appeared to have less influence on ANC uptake if they did not live in the same household as their daughters-in-law [53] (Tables 2 and 3).

**Table 3:** Synthesis of qualitative findings on the role of social capital on maternal and child health services uptake in LMICs

Findings and reflections	Category	Synthesized findings
Finding: Across network support patterns, most women indicated that network members were showed empathy in their interactions with them. ***Reflection***: *“I have a friend here from my native place; with her only I am sharing my feelings.” (26 year old, high school educated) Raman et al., page 132* ***Reflection***: *“we are free and open with each other…Cecilia too shows me love [inaudible] so she chats with me about things that will bring laughter” ( Woman, Student, 19 yrs.) Cofie et al., page 8* ***Reflection***: *“I was telling her not to worry, but I myself was worried deep inside.” (32-year-old farmer).* *Sapkota et al., page 48*	Emotional support	**Social supports enabled women to use MCH care.**
Finding: Network members visited frequently during her pregnancy. ***Reflection***: *“When I am not able to go visit her [mother], she comes to visit me and asks about me, that made me know she loves me” (Woman, Student, 19 yrs.) Cofie et al., page 8*		
Finding: Religious leaders provide emotional and spiritual support to their followers. ***Reflection***: *“In the Orthodox Church while the religious leaders pray, they also pray for pregnant women and for women in postpartum period” (Female FGD participant from Gomma district) Mamo et al., page 7*		
Finding: Most women received advice to use ANC and health facility delivery care. ***Reflection***: *“The public hearing generates the awareness of the public that pregnancy is not a very simple thing …that they have to come to the facilities for the technical support.” (PGO-1) Papp et al., page 455* ***Reflection***: *“The role of the mosque is broad; the first one is advising the community to believe what health professionals order us to do is essential you die if you are ordered to die and live if you are ordered to live by God but you should believe the advice given by health professionals is true.” (Muslim religious leader from Seka district) Mamo et al., page 8*	Informational support	
Finding: Family members, particularly Mother in laws[MIL], mothers and grandmothers advice women and provides suggestions on how to experience safe pregnancy and delivery ***Reflection***: *“My mother had 11 children, out of which seven surviving… therefore she gave all advice (during pregnancy). And I followed her advice.” (32 years, working woman) Raman et al., page 132* ***Reflection***: *“Not lift heavy things…eat well”( FGDs with MILs) Cofie et al., page 8*		
Finding: Network members lived in close proximity provide advice to the pregnant women ***Reflection***: *“Hamdia (brother-in-law’s wife). . .and my husband. They told me to not work as hard as I used to because now that I am pregnant I need to be cautious of the kind of work that I do. .Bintu (Friend) was also involved. .if I was not feeling well, I would call her and tell her. She would then tell me to go to the hospital because that is where we will find out what is actually wrong with me. . .”( woman in the facility birth group) Cofie et al., page 8*		
Finding: Close network members were ready to avail a transport vehicle for travelling to health facility during child birth. ***Reflection***: *“When her husband is not there. .you [MIL] would then talk to any family member available at that time, for that person to look for a motorbike, fuel it and take her to hospital” (FGD, MIL) Cofie et al., page 9* ***Reflection***: *“My auntie helped in supporting my wife to sit on the motorbike. My auntie sat at the back as we took my wife to the clinic”. [Husband, Farmer, 27 yrs.] Cofie et al., page 9*	Instrumental support	
Finding: Network members able to help her access and utilize facility delivery ***Reflection***: *“The women health development army exists in the form of AFOSHA (cultural self-help system). They help one another not only when someone dies, but also when someone gives birth and during festivals. They even help one another financially. They contribute money and buy basic household materials.” (Male FGD participant from Gomma district) Mamo et al., page 6*		
Finding: Some women had numerous sources of support, their own mothers, female relatives and friends. ***Reflection: “****There are enough people around me to talk to and support, (but) mainly I would tell my mother about everything. She has been very supportive throughout.” (29 years, educated) Raman et al., page 132* ***Reflection***: *“When I was vomiting for the first few months, three different friends used to cook different dishes for me every day; they looked after me so well.” (27 year old, nuclear family) Raman et al., page 133*		
Finding: MDA and WDA leaders are good in passing different knowledge to mothers and members of the community during community meetings, women’s association meetings, antenatal outreach sessions, and coffee ceremony ***Reflection***: *“We are doing many activities like advising women to prepare before delivery. There could be different problems during delivery and they may need money, so I inform them to save certain money and keep it with them.” (MDA from Gomma district) Mamo et al., page 6*	Promotion of MCH services	**Receiving health information from trusted people enhanced uptake of MCH services.**
Finding: HEWs, WDA and religious leaders are also participating on community mobilization activities including use of full ANC services, health facility delivery and PNC ***Reflection***: *“What I should do for pregnant women during pregnancy is taking them to health facility for regular check-up and helping them to go and deliver in health facility and taking the children to health facility for vaccination.” (Muslim Religious leader from Kersa district) Mamo et al., page 6*		
Finding: Assistance with community, husbands and WDA support women during and after pregnancy period. ***Reflection***: *“…when she is ready to deliver, I will take her to the health center and then come back home with her… after delivery I am responsible for preparing food and giving her advice about not working beyond her capacity and for washing the baby clothes.” (WDA leader from Seka district) Mamo et al., page 7* ***Reflection***: *“My son is always behind his wife. He is not only helping, but also supporting her all the time.” (Mother-in-law 3) Simkhada et al. page 7*	Provision of continuous support	
Finding: Integrating activities between community leaders to be enhance strong relationship and communication between HEWs, primary health care units and community members ***Reflection***: *“What makes women health development army leader support special is that, they involve starting by enrolling the pregnant women and reporting to health extension worker at the termination of first menstrual cycle”(HEWs from Kersa district) Mamo et al., page 8*	A link between communities and health system	
Findings: Some members of the community cannot go to the hospital for health care services for whatever problem without first going to herbalists. Ill health is as a result of evil spirits and traditional systems of health care were best-placed to deal with them ***Reflection: “…****in the olden days of our grandfathers and grandmothers, we just used to stay like that when a woman got pregnant; she would just use some roots (herbs) and she would deliver without any problem.****”****(Female FGD participant, 63 years, Married, 10 children, Viphalani village) Mochache et al., page 4*	Influence of socio-cultural norms	**Socio-cultural factors influenced uptake MCH services**
Finding: Women prefer facility delivery if complications arise during the birthing process ***Reflection***: *“They [women] did not easily go to the hospital or clinics, furthermore when a woman got pregnant, she just stayed at home … and in case of any complications there was always traditional means of treatment. Certain plants were used to relieve women of abdominal pains and it has really taken long to change. ” (Male FGD participant, 50 years, Married, 3 children, Magodzoni village) Mochache et al., page 5*		
Finding: In the culture of some community, a woman has to stay indoors for a long period of time without accessing MCH services ***Reflection***: *“In my community, a woman has to stay indoors [for a month to 40 days] until the baby’s skin lightness disappears, that’s when you get out (local phrase used: ‘mpaka mtoto afunike jua’).” (Female IDI participant, 28 years, Married, 1 child, Kwale town) Mochache et al., page 5*		
Findings: Maternal figures play a critical role in the decision-making pathway for choice of place of delivery. Some network members tended to first seek the involvement of a traditional birth attendant (TBA) during women’s labor and did not make timely arrangements to transport women to a facility. ***Reflection***: *“My mother-in-law said that pregnant women didn’t go for antenatal check-ups in the old days. She told me that she had all her children without any antenatal check-ups and all are fine. She questioned why different foods and antenatal check-ups are necessary for pregnant women. That’s why I didn’t go” (Non-user Woman 1). Simkhada et al., page 5* ***Reflection***: *“she told me to wait for a while because she was going call Esi Eyeh [TBA] for her to come and check whether my pregnancy was due.” (woman, Trader, 20 yrs.) Cofie et al., page 10* ***Reflection***: “*Long time ago, our grandmothers, even our mothers, if a woman was in labor, the father would say we should wait first, and the mother would take charge … and that time, people never went for clinic, they did not know if the load they were carrying [pregnancy] was safe or not.” (Female IDI participant, 28 years, Married, 4 children, Mtsamviani village) Mochache et al., page 6* *“I assisted my first daughter to deliver, I also assisted my sister in-law, my granddaughter … I am not [a TBA], but I thank God …” (Female FGD participant, 46 years, Married, 7 children, Viphalani village) Mochache et al., page 6* ***Reflection***: *“we only take women to hospital if the mkunga [TBA] has failed, that is what I always see … Nobody will take a woman to hospital at the onset of labor, and if you hear a woman has been taken there (hospital), then it is because the mkunga has failed … if you realize as per the woman’s condition she can deliver on her own, because most of the time she delivers at home, you don’t have to go to the hospital. You can bring the mkunga and she will deliver the baby.” (Male FGD participant, 43 years, Married, 2 children, Kiruku village) Mochache et al., page 6* ***Reflection***: *“I will ask my husband first … then he will find out what his mother thinks. After that, we will do what his mother says …” (Female IDI participant, 23 years, Married, 2 children, Kwale town) Mochache et al., page 7*	Role of significant matriarchal figure	
Finding: Religious norms influence women’s decision making on the use of MCH services. Women would avoid seeking a health facility delivery service if no female provider was available ***Reflection***: *“Religion says that, but those who are employed there [at the hospitals] have the experience required to serve both men and women. You cannot force the government and say that you only want female employees at the hospital. So, it is true religion refuses (sic) us, but if you get to the hospital with a woman in labor, you cannot choose and insist that you want only a woman to attend to your wife. You must accept the service to be given by anybody”. (Male FGD participant, 42 years, Married, 2 wives, 5 children, Mkoyo) Mochache et al., page 7*	Influence of religious norms	
Finding: Islam religious norm might forbid women from being seen by other men except their husbands. ***Reflection***: “*In the Digo way of life, they are all Muslims and religion has refused (sic) us and says women must only be assisted by fellow women during child delivery.” (Male FGD participant, 51 years, Married, 8 children, Mkoyo sub-location) Mochache et al., page 6*		
Finding: The role of a woman in this community was mainly to give birth and have many children ***Reflection***: *“The pregnancy is yours, I′ m only waiting for the babies; my role as a husband is for the wife to inform me when there is no flour in the house and I provide, that is all.” (Male FGD participant, 70 years, Married, 11 children, serves as the local sheikh, Kifuku village) Mochache et al., page 7*	Role of gender stereotypes	
Finding: Gender-related power imbalances in decision making related to MCH services. ***Reflection***: “*There is male dominancy all over India. The pregnant lady is not able to take her decision individually. She has to depend upon her mother-in-law, her husband or the society itself. This decision-making is also very late and they are coming to the institution very late. In spite of all efforts done by the ASHA, pregnant women are not getting the effective maternal check-ups and early transportation.” (HP-1) Papp et al., page 456* ***Reflection***: *“If it is a matter of giving birth you have already done so. What is this business of you going to the health facility every other time? You must be having an affair with someone there.” (Female FGD participant, 63 years, Married, 10 children, Viphalani village) Mochache et al., page 7* ***Reflection***: *“He wants four children, not me. He himself said he wants four. From his opinion he does not want me to use family planning.” (Female IDI participant, 23 years, Married, 1 child, Simkumbe village) Mochache et al., page 7*		

*ANC:* Antenatal Care, *FGD:* Focus Group discussion, *HEWs:* Health Extension Workers, *HP*: Health Provider, *IDI:* In-Depth Interview, *MCH:* Maternal and Child Health, *MDA:* Male Development Army, *MIL:* Mother In Laws, *PGO*: Policy-makers/Government Officials, *PNC:* Postnatal Care, *TBA*: Traditional Birth Attendant, *WDA*: Women Development Army

## Discussion

This study aimed to synthesize the available literature about the role of social capital on the uptake of MCH services in LMICs. The results of this study indicated that social capital has dual roles with the favorable impact to improve MCH services uptake but also some negative consequences. Network members who have sufficient health information provide advice to the pregnant women to access ANC, avail a transport vehicle for travelling to health facility during childbirth. In contrast, some network members who lived in close proximity tended to first seek the involvement of a traditional birth attendant during women’s labor and did not make timely arrangements to transport women to a facility. These findings are in line with other studies that have found a significant effect of social capital on other health outcomes [69, 70].

Women’s social capital had great contribution on the uptake of MCH services. Women from villages with a self-help group were more likely to give birth in a health facility [46]. Likewise, the qualitative component of this review complemented that family members, particularly husbands, mother in laws, mothers and grandmothers advice women provided suggestions on how to experience safe pregnancy and health facility delivery [51].

Women who lived in communities with higher social capital had higher odds of ANC uptake [27]. Most women received support from group members to use facility-based pregnancy and delivery care [50]. Besides male partners’ involvement in MCH care has benefits on better uptake MCH services [47]. This might be that husbands who attend ANC with their partners acquire useful knowledge on how to support their wives during pregnancy. In line with this finding, a systematic review in developing countries revealed that good male involvement improved the uptake of MCH services [71].

Social capital influences uptake of MCH services through social networks between communities or community members and representatives of formal institutions such as health care providers, teachers and government officers [72]. Moreover, involvement of religious leaders, health extension workers, women developmental army leaders, and selected community members could enhance use of MCH services. Women who received health information from people they trust are more likely to access and use health services [48]. Neighborhoods with higher levels of social trust experience lower rates of health and health related problems, and have fewer signs of physical disorder, making residents of these neighborhoods feel safer [73–76].

Socio-cultural factors might hinder uptake of MCH services [52]. In some communities, women who gave birth have to stay indoors for a month up to 40 days; some members of the community cannot seek health care services at health facilities for whatever problems without first going to herbalists; only opting for a facility delivery if complications arise during the birthing process and traditional birth attendants play a critical role in the decision-making pathway for choice of place of delivery [48, 52, 53]. In line with these findings, a previous systematic review reported that influence of traditional beliefs and sociocultural norms was high during childbirth. Women interpreted their expectations through the lens of family birth stories and social norms [77].

There is no single universally accepted measurement tool for social capital that may partly attributed to the fact that the nature of social networks as social interactions and functions could be context specific. Most of the tools used to measure social capital in LMICs were originally developed in higher income countries. Cultural adaptation of the tool is an important procedure especially when the original tool is from a different cultural setting [78, 79]. However, the review identified that social capital was measured at both at individual and at community levels. Assessment of social capital at individual or community level may depend upon researchers’ interest. Community level measures are required if the researcher is aimed to examine contextual outcomes. When interpreting aggregated social capital at group level, aggregation of individual social capital measures would not resemble the social capital of the whole community [56–59, 61–66, 80–85]. Most studies included in this review assessed both structural and cognitive social capital in which it was consistent with other systematic reviews conducted in least developed countries and LMICs [78, 79, 86, 87].

## Strengths and limitations of the review

The current review has its own strength and limitations. Its strength includes its ability to integrate the results of quantitative and qualitative studies. Regarding the limitations, there is no accepted definition and uniform measurement tool for social capital across the studies and thus was not possible to conduct meta-analysis. Also, some studies have no information about the validity and reliability of social capital measurement tool. Although our searching was comprehensive, it is possible that some articles were missed. Moreover, studies with statistically significant findings are more likely to be published. Due to this reason, some unpublished studies are missed that results publication bias.

### Implication for public policy makers and researchers

The evidence in this review showed that social capital may contribute for improving women’s uptake of MCH services for themselves and their children. Therefore, it will be worthwhile for policy makers to design strategies on strengthening community’s social capital to enhance uptake of MCH services. It may also be necessary for available social networks and groups to be targeted for health education and promotion activities.

## Conclusions

Social capital has great contribution on uptake of MCH services even though socio-cultural factors may influence its functionality. Countries aiming at improving MCH services can be benefited from adapting existing context-specific social networks in the community. This review identified limited available evidence examining the role of social capital on MCH services uptake. Measurement tools for social capital have no uniformity across studies and most of them were conducted using a cross-sectional design. Hence, future studies may be required for in-depth understanding of how social capital could improve MCH services.

## Supplementary information

**Additional file 1**

**Additional file 2**

**Additional file 3**

## Data Availability

The datasets used and/or analyzed during the current study are available from the corresponding author on reasonable request.

## Abbreviations

ANC: Antenatal Care
FGDs: Focus Group Discussions
IDI: In-Depth Interviews
JBI: Joanna Briggs Institute
LMICs: Low- and Middle-Income Countries
MCH: Maternal and Child Health Services
PNC: Postnatal Care
PRISMA: Preferred Reporting Items for Systematic Reviews and Meta-Analyses
SDGs: Sustainable developmental goals
