# Supplementary material for: Social capital and maternal and child health services uptake in low- and middle-income countries: mixed methods systematic review
Source: BMC Health Serv Res. 2021 Oct 22;21:1142. doi: 10.1186/s12913-021-07129-1 (PMC8539777; doi:10.1186/s12913-021-07129-1)
Supplement: Supplementary file 2 — Additional file 2 [file 12913_2021_7129_MOESM2_ESM.docx]

**Supplementary file 2.** Search strategy

| Social capital(domain1) | Maternal and child health services(domain 2 = A OR B OR C OR D) | | | | Low- and middle- income countries(domain 3) |
| --- | --- | --- | --- | --- | --- |
|  | Maternal and child health services(A) | ANC(B) | Institutional delivery(C) | PNC(D) |  |
| **PubMed** | | | | | |
| (("social capital"[MeSH Terms] OR "social capital"[Title/Abstract] OR "social support"[MeSH Terms] OR "social trust"[Title/Abstract] OR "social network"[Title/Abstract] OR "social support networks"[Title/Abstract] OR "community networks"[Title/Abstract] OR "community care networks"[Title/Abstract] OR "community health networks"[Title/Abstract] OR "neighborhood cohesion"[Title/Abstract] OR "informal social control"[Title/Abstract] OR "collective efficacy"[Title/Abstract] OR "civil society"[Title/Abstract] OR "group participation"[Title/Abstract] OR "social relationships"[Title/Abstract] OR "social cohesion"[Title/Abstract]) AND (((("maternal health services"[MeSH Terms] OR "maternal health service"[Title/Abstract] OR "maternal health care"[Title/Abstract] OR "maternal child health services"[Title/Abstract]) OR ("prenatal care"[MeSH Terms] OR "prenatal care"[Title/Abstract] OR "antenatal care"[Title/Abstract])) OR ("health facility delivery"[Title/Abstract] OR "facility delivery"[Title/Abstract] OR "skilled birth"[Title/Abstract] OR "skilled delivery"[Title/Abstract] OR "skilled birth delivery"[Title/Abstract] OR "facility based delivery"[Title/Abstract] OR "institutional delivery"[Title/Abstract] OR (("Health"[MeSH Terms] OR "Health"[All Fields] OR "health s"[All Fields] OR "healthful"[All Fields] OR "healthfulness"[All Fields] OR "healths"[All Fields]) AND "center birth"[Title/Abstract]) OR "hospital birth"[Title/Abstract] OR "hospital delivery"[Title/Abstract])) OR ("postnatal care"[MeSH Terms] OR "postnatal care"[Title/Abstract] OR "postpartum care"[Title/Abstract] OR "postpartum programs"[Title/Abstract] OR "postpartum program"[Title/Abstract]))) AND ("Africa"[Mesh] OR "Asia"[Mesh] OR Afghanistan*[tiab] OR Albania*[tiab] OR Algeria*[tiab] OR American Samoa*[tiab] OR Angola*[tiab] OR Argentina*[tiab] OR Armenia*[tiab] OR Azerbaijan*[tiab] OR Bangladesh*[tiab] OR Belarus*[tiab] OR Belize*[tiab] OR Benin*[tiab] OR Bhutan*[tiab] OR Bolivia*[tiab] OR Bosnia and Herzegovina*[tiab] OR Botswana*[tiab] OR Brazil*[tiab] Bulgaria*[tiab] OR Burkina Faso*[tiab] OR Burundi*[tiab] OR Cabo Verde*[tiab] OR Cambodia*[tiab] OR Cameroon*[tiab] OR Central African Republic*[tiab] OR Chad*[tiab] OR China*[tiab] OR Colombia*[tiab] OR Comoros*[tiab] OR Congo Dem. Rep.*[tiab] OR Congo Rep.*[tiab] OR Costa Rica*[tiab] OR Côte d'Ivoire*[tiab] OR Cuba*[tiab] OR Djibouti*[tiab] OR Dominica*[tiab] OR Dominican Republic*[tiab] OR Ecuador*[tiab] OR Egypt*[tiab] OR El Salvador*[tiab] OR Equatorial Guinea*[tiab] OR Eritrea*[tiab] OR Eswatini*[tiab] OR Ethiopia*[tiab] OR Fiji*[tiab] OR Gabon*[tiab] OR Gambia*[tiab] OR Georgia*[tiab] OR Ghana*[tiab] OR Grenada*[tiab] OR Guatemala*[tiab] OR Guinea*[tiab] OR Guinea-Bissau*[tiab] OR Guyana*[tiab] OR Haiti*[tiab] OR Honduras*[tiab] OR India*[tiab] OR Indonesia*[tiab] OR Iran*[tiab] OR Iraq*[tiab] OR Jamaica*[tiab] OR Jordan*[tiab] OR Kazakhstan*[tiab] OR Kenya*[tiab] OR Kiribati*[tiab] OR Korea Dem. People's Rep.*[tiab] OR Kosovo*[tiab] OR Kyrgyz Republic*[tiab] OR Lao PDR*[tiab] OR Lebanon*[tiab] OR Lesotho*[tiab] OR Liberia*[tiab] OR Libya*[tiab] OR Madagascar*[tiab] OR Malawi*[tiab] OR Malaysia*[tiab] OR Maldives*[tiab] OR Mali*[tiab] OR Marshall Islands*[tiab] OR Mauritania*[tiab] OR Mexico*[tiab] OR Micronesia Fed. Sts.*[tiab] OR Moldova*[tiab] OR Mongolia*[tiab] OR Montenegro*[tiab] OR Morocco*[tiab] OR Mozambique*[tiab] OR Myanmar*[tiab] OR Namibia*[tiab] OR Nepal*[tiab] OR Nicaragua*[tiab] OR Niger*[tiab] OR Nigeria*[tiab] OR North Macedonia*[tiab] OR Pakistan*[tiab] OR Papua New Guinea*[tiab] OR Paraguay*[tiab] OR Peru*[tiab] OR Philippines*[tiab] OR Russian Federation*[tiab] OR Rwanda*[tiab] OR Samoa*[tiab] OR São Tomé and Principe*[tiab] OR Senegal*[tiab] OR Serbia*[tiab] OR Sierra Leone*[tiab] OR Solomon Islands*[tiab] OR Somalia*[tiab] OR South Africa*[tiab] OR South Sudan*[tiab] OR Sri Lanka*[tiab] OR St. Lucia*[tiab] OR St. Vincent and the Grenadines*[tiab] OR Sudan*[tiab] OR Suriname*[tiab] OR Syrian Arab Republic*[tiab] OR Tajikistan*[tiab] OR Tanzania*[tiab] OR Thailand*[tiab] OR Timor-Leste*[tiab] OR Togo*[tiab] OR Tonga*[tiab] OR Tunisia*[tiab] OR Turkey*[tiab] OR Turkmenistan*[tiab] OR Tuvalu*[tiab] OR Uganda*[tiab] OR Ukraine*[tiab] OR Uzbekistan*[tiab] OR Vanuatu*[tiab] OR Venezuela RB*[tiab] OR Vietnam*[tiab] OR West Bank and Gaza*[tiab] OR Yemen Rep.*[tiab] OR Zambia*[tiab] OR Zimbabwe*[tiab]) | | | | | |
| "social capital"[MeSH Terms] OR "social capital"[Title/Abstract] OR "social support"[MeSH Terms] OR "social trust"[Title/Abstract] OR "social network"[Title/Abstract] OR "social support networks"[Title/Abstract] OR "community networks"[Title/Abstract] OR "community care networks"[Title/Abstract] OR "community health networks"[Title/Abstract] OR "neighborhood cohesion"[Title/Abstract] OR "informal social control"[Title/Abstract] OR "collective efficacy"[Title/Abstract] OR "civil society"[Title/Abstract] OR "group participation"[Title/Abstract] OR "social relationships"[Title/Abstract] OR "social cohesion"[Title/Abstract] | "maternal health services"[MeSH Terms] OR "maternal health service"[Title/Abstract] OR "maternal health care"[Title/Abstract] OR "maternal child health services"[Title/Abstract] | "prenatal care"[MeSH Terms] OR "prenatal care"[Title/Abstract] OR "antenatal care"[Title/Abstract] | "health facility delivery"[Title/Abstract] OR "facility delivery"[Title/Abstract] OR "skilled birth"[Title/Abstract] OR "skilled delivery"[Title/Abstract] OR "skilled birth delivery"[Title/Abstract] OR "facility based delivery"[Title/Abstract] OR "institutional delivery"[Title/Abstract] OR (("Health"[MeSH Terms] OR "Health"[All Fields] OR "health s"[All Fields] OR "healthful"[All Fields] OR "healthfulness"[All Fields] OR "healths"[All Fields]) AND "center birth"[Title/Abstract]) OR "hospital birth"[Title/Abstract] OR "hospital delivery"[Title/Abstract] | "postnatal care"[MeSH Terms] OR "postnatal care"[Title/Abstract] OR "postpartum care"[Title/Abstract] OR "postpartum programs"[Title/Abstract] OR "postpartum program"[Title/Abstract] | "Africa"[Mesh] OR "Asia"[Mesh] OR Afghanistan*[tiab] OR Albania*[tiab] OR Algeria*[tiab] OR American Samoa*[tiab] OR Angola*[tiab] OR Argentina*[tiab] OR Armenia*[tiab] OR Azerbaijan*[tiab] OR Bangladesh*[tiab] OR Belarus*[tiab] OR Belize*[tiab] OR Benin*[tiab] OR Bhutan*[tiab] OR Bolivia*[tiab] OR Bosnia and Herzegovina*[tiab] OR Botswana*[tiab] OR Brazil*[tiab] Bulgaria*[tiab] OR Burkina Faso*[tiab] OR Burundi*[tiab] OR Cabo Verde*[tiab] OR Cambodia*[tiab] OR Cameroon*[tiab] OR Central African Republic*[tiab] OR Chad*[tiab] OR China*[tiab] OR Colombia*[tiab] OR Comoros*[tiab] OR Congo Dem. Rep.*[tiab] OR Congo Rep.*[tiab] OR Costa Rica*[tiab] OR Côte d'Ivoire*[tiab] OR Cuba*[tiab] OR Djibouti*[tiab] OR Dominica*[tiab] OR Dominican Republic*[tiab] OR Ecuador*[tiab] OR Egypt*[tiab] OR El Salvador*[tiab] OR Equatorial Guinea*[tiab] OR Eritrea*[tiab] OR Eswatini*[tiab] OR Ethiopia*[tiab] OR Fiji*[tiab] OR Gabon*[tiab] OR Gambia*[tiab] OR Georgia*[tiab] OR Ghana*[tiab] OR Grenada*[tiab] OR Guatemala*[tiab] OR Guinea*[tiab] OR Guinea-Bissau*[tiab] OR Guyana*[tiab] OR Haiti*[tiab] OR Honduras*[tiab] OR India*[tiab] OR Indonesia*[tiab] OR Iran*[tiab] OR Iraq*[tiab] OR Jamaica*[tiab] OR Jordan*[tiab] OR Kazakhstan*[tiab] OR Kenya*[tiab] OR Kiribati*[tiab] OR Korea Dem. People's Rep.*[tiab] OR Kosovo*[tiab] OR Kyrgyz Republic*[tiab] OR Lao PDR*[tiab] OR Lebanon*[tiab] OR Lesotho*[tiab] OR Liberia*[tiab] OR Libya*[tiab] OR Madagascar*[tiab] OR Malawi*[tiab] OR Malaysia*[tiab] OR Maldives*[tiab] OR Mali*[tiab] OR Marshall Islands*[tiab] OR Mauritania*[tiab] OR Mexico*[tiab] OR Micronesia Fed. Sts.*[tiab] OR Moldova*[tiab] OR Mongolia*[tiab] OR Montenegro*[tiab] OR Morocco*[tiab] OR Mozambique*[tiab] OR Myanmar*[tiab] OR Namibia*[tiab] OR Nepal*[tiab] OR Nicaragua*[tiab] OR Niger*[tiab] OR Nigeria*[tiab] OR North Macedonia*[tiab] OR Pakistan*[tiab] OR Papua New Guinea*[tiab] OR Paraguay*[tiab] OR Peru*[tiab] OR Philippines*[tiab] OR Russian Federation*[tiab] OR Rwanda*[tiab] OR Samoa*[tiab] OR São Tomé and Principe*[tiab] OR Senegal*[tiab] OR Serbia*[tiab] OR Sierra Leone*[tiab] OR Solomon Islands*[tiab] OR Somalia*[tiab] OR South Africa*[tiab] OR South Sudan*[tiab] OR Sri Lanka*[tiab] OR St. Lucia*[tiab] OR St. Vincent and the Grenadines*[tiab] OR Sudan*[tiab] OR Suriname*[tiab] OR Syrian Arab Republic*[tiab] OR Tajikistan*[tiab] OR Tanzania*[tiab] OR Thailand*[tiab] OR Timor-Leste*[tiab] OR Togo*[tiab] OR Tonga*[tiab] OR Tunisia*[tiab] OR Turkey*[tiab] OR Turkmenistan*[tiab] OR Tuvalu*[tiab] OR Uganda*[tiab] OR Ukraine*[tiab] OR Uzbekistan*[tiab] OR Vanuatu*[tiab] OR Venezuela RB*[tiab] OR Vietnam*[tiab] OR West Bank and Gaza*[tiab] OR Yemen Rep.*[tiab] OR Zambia*[tiab] OR Zimbabwe*[tiab] |
| **Scopus** | | | | | |
| ( TITLE-ABS ( "social capital" OR "social capital" OR "social support" OR "social trust" OR "social network" OR "social support networks" OR "community networks" OR "community care networks" OR "community health networks" OR "neighborhood cohesion" OR "informal social control" OR "collective efficacy" OR "civil society" OR "group participation" OR "social relationships" OR "social cohesion" ) ) AND ( ( TITLE-ABS ( "maternal health services" OR "maternal health care" OR "maternal child health services" ) ) OR ( TITLE-ABS ( "prenatal care" OR "antenatal care" ) ) OR ( TITLE-ABS ( "health facility delivery" OR "facility delivery" OR "skilled birth" OR "skilled delivery" OR "skilled birth delivery" OR "facility based delivery" OR "institutional delivery" OR "Health center birth" OR "hospital birth" OR "hospital delivery" ) ) OR ( TITLE-ABS ( "postnatal care" OR "postpartum care" OR "postpartum programs" OR "postpartum program" ) ) ) AND ( TITLE-ABS ( "Africa" OR "Asia" OR afghanistan OR albania OR algeria OR "American Samoa" OR angola OR argentina OR armenia OR azerbaijan OR bangladesh OR belarus OR belize OR benin OR bhutan OR bolivia OR "Bosnia and Herzegovina" OR botswana OR brazil OR bulgaria OR "Burkina Faso" OR burundi OR "Cabo Verde" OR cambodia OR cameroon OR "Central African Republic" OR chad OR china OR colombia OR comoros OR "Congo Dem. Rep." OR "Congo Rep. OR Costa Rica" OR "Côte d'Ivoire" OR cuba OR djibouti OR dominica OR "Dominican Republic" OR ecuador OR egypt OR "El Salvador" OR "Equatorial Guinea" OR eritrea OR eswatini OR ethiopia OR fiji OR gabon OR gambia OR georgia OR ghana OR grenada OR guatemala OR guinea OR "Guinea-Bissau" OR guyana OR haiti OR honduras OR india OR indonesia OR iran OR iraq OR jamaica OR jordan OR kazakhstan OR kenya OR kiribati OR "Korea Dem. People's Rep." OR "Kosovo" OR "Kyrgyz Republic" OR "Lao PDR" OR lebanon OR lesotho OR liberia OR libya OR madagascar OR malawi OR malaysia OR maldives OR mali OR "Marshall Islands" OR mauritania OR mexico OR "Micronesia Fed. Sts." OR moldova OR mongolia OR montenegro OR morocco OR mozambique OR myanmar OR namibia OR nepal OR nicaragua OR niger OR nigeria OR "North Macedonia" OR pakistan OR "Papua New Guinea" OR paraguay OR peru OR philippines OR "Russian Federation" OR rwanda OR samoa OR "São Tomé and Principe" OR senegal OR serbia OR "Sierra Leone" OR "Solomon Islands" OR somalia OR "South Africa" OR "South Sudan" OR "Sri Lanka" OR "St. Lucia" OR "St. Vincent and the Grenadines" OR sudan OR suriname OR "Syrian Arab Republic" OR tajikistan OR tanzania OR thailand OR "Timor-Leste" OR togo OR tonga OR tunisia OR turkey OR turkmenistan OR tuvalu OR uganda OR ukraine OR uzbekistan OR vanuatu OR "Venezuela RB" OR vietnam OR "West Bank and Gaza" OR "Yemen Rep." OR zambia OR zimbabwe ) ) | | | | | |
| TITLE-ABS ( "social capital" OR "social capital" OR "social support" OR "social trust" OR "social network" OR "social support networks" OR "community networks" OR "community care networks" OR "community health networks" OR "neighborhood cohesion" OR "informal social control" OR "collective efficacy" OR "civil society" OR "group participation" OR "social relationships" OR "social cohesion") | TITLE-ABS ( "maternal health services" OR "maternal health care" OR "maternal child health services" ) | TITLE-ABS ( "prenatal care" OR "antenatal care" ) | TITLE-ABS ( "health facility delivery" OR "facility delivery" OR "skilled birth" OR "skilled delivery" OR "skilled birth delivery" OR "facility based delivery" OR "institutional delivery" OR "Health center birth" OR "hospital birth" OR "hospital delivery" ) | TITLE-ABS ( "postnatal care" OR "postpartum care" OR "postpartum programs" OR "postpartum program" ) | TITLE-ABS ("Africa" OR "Asia" OR Afghanistan OR Albania OR Algeria OR "American Samoa" OR Angola OR Argentina OR Armenia OR Azerbaijan OR Bangladesh OR Belarus OR Belize OR Benin OR Bhutan OR Bolivia OR "Bosnia and Herzegovina" OR Botswana OR Brazil OR Bulgaria OR "Burkina Faso" OR Burundi OR "Cabo Verde" OR Cambodia OR Cameroon OR "Central African Republic" OR Chad OR China OR Colombia OR Comoros OR "Congo Dem. Rep." OR "Congo Rep. OR Costa Rica" OR "Côte d'Ivoire" OR Cuba OR Djibouti OR Dominica OR "Dominican Republic" OR Ecuador OR Egypt OR "El Salvador" OR "Equatorial Guinea" OR Eritrea OR Eswatini OR Ethiopia OR Fiji OR Gabon OR Gambia OR Georgia OR Ghana OR Grenada OR Guatemala OR Guinea OR "Guinea-Bissau" OR Guyana OR Haiti OR Honduras OR India OR Indonesia OR Iran OR Iraq OR Jamaica OR Jordan OR Kazakhstan OR Kenya OR Kiribati OR "Korea Dem. People's Rep." OR "Kosovo" OR "Kyrgyz Republic" OR "Lao PDR" OR Lebanon OR Lesotho OR Liberia OR Libya OR Madagascar OR Malawi OR Malaysia OR Maldives OR Mali OR "Marshall Islands" OR Mauritania OR Mexico OR "Micronesia Fed. Sts." OR Moldova OR Mongolia OR Montenegro OR Morocco OR Mozambique OR Myanmar OR Namibia OR Nepal OR Nicaragua OR Niger OR Nigeria OR "North Macedonia" OR Pakistan OR "Papua New Guinea" OR Paraguay OR Peru OR Philippines OR "Russian Federation" OR Rwanda OR Samoa OR "São Tomé and Principe" OR Senegal OR Serbia OR "Sierra Leone" OR "Solomon Islands" OR Somalia OR "South Africa" OR "South Sudan" OR "Sri Lanka" OR "St. Lucia" OR "St. Vincent and the Grenadines" OR Sudan OR Suriname OR "Syrian Arab Republic" OR Tajikistan OR Tanzania OR Thailand OR "Timor-Leste" OR Togo OR Tonga OR Tunisia OR Turkey OR Turkmenistan OR Tuvalu OR Uganda OR Ukraine OR Uzbekistan OR Vanuatu OR "Venezuela RB" OR Vietnam OR "West Bank and Gaza" OR "Yemen Rep." OR Zambia OR Zimbabwe) |

Search ended on January 22, 2021
