# Supplementary material for: Social capital and maternal and child health services uptake in low- and middle-income countries: mixed methods systematic review
Source: BMC Health Serv Res. 2021 Oct 22;21:1142. doi: 10.1186/s12913-021-07129-1 (PMC8539777; doi:10.1186/s12913-021-07129-1)
Supplement: Supplementary file 3 — Additional file 3 [file 12913_2021_7129_MOESM3_ESM.docx]

**Supplementary file 3.** Methodological quality assessment

| **First author, year of publication and reference number** | **Q1** | **Q2** | **Q3** | **Q4** | **Q5** | **Q6** | **Q7** | **Q8** | **Q9** | **Q10** | **Overall quality of the study** |
| --- | --- | --- | --- | --- | --- | --- | --- | --- | --- | --- | --- |
| Critical appraisal for included quantitative studies | | | | | | | | | | | |
| Singh et al., 2014[38] | Y | Y | Y | Y | U | N | Y | Y |  |  | 6(Moderate) |
| Story et al., 2014[24] | Y | Y | Y | Y | U | N | Y | Y |  |  | 6(Moderate) |
| Semali et al., 2015[39] | Y | Y | Y | Y | Y | N | Y | Y |  |  | 7(High) |
| Saha et al., 2013[45] | Y | Y | Y | Y | U | N | Y | Y |  |  | 6(Moderate) |
| Mohammed et al., 2019[46] | N | Y | Y | Y | N | N | Y | Y |  |  | 5(Moderate) |
| McTavish et al., 2015[40] | N | Y | Y | Y | U | N | Y | Y |  |  | 5(Moderate) |
| **Number of studies that achieved compliance** | 4 | 8 | 8 | 8 | 1 | 0 | 8 | 8 |  |  |  |
| Critical appraisal for included qualitative studies | | | | | | | | | | | |
| Cofie et al., 2018[49] | N | Y | Y | Y | Y | N | N | Y | Y | Y | 7(High) |
| Mochache et al., 2020[50] | N | Y | Y | Y | Y | Y | N | Y | Y | Y | 8(High) |
| Papp et al., 2013[47] | N | Y | Y | Y | Y | N | N | Y | N | Y | 6(Moderate) |
| Raman et al., 2014[48] | N | Y | Y | Y | Y | Y | N | Y | Y | Y | 8(High) |
| Mamo et al., 2019[33] | N | Y | Y | Y | Y | Y | N | Y | Y | Y | 8(High) |
| Simkhada et al., 2010[52] | N | Y | Y | Y | Y | Y | N | Y | Y | Y | 8(High) |
| Sapkota et al., 2012[53] | N | Y | Y | Y | Y | N | N | Y | Y | Y | 7(High) |
| **Number of studies that achieved compliance** | 0 | 7 | 7 | 7 | 7 | 5 | 0 | 7 | 6 | 7 |  |

Criteria were adapted from the JBI Critical Appraisal Checklist for descriptive/case series research[82]. **For quantitative studies**: (1) Was the study based on a random or pseudo-random sample? (2) Were the criteria for inclusion in the sample clearly defined? (3) Were confounding factors identified and strategies to deal with them stated? (4) Were outcomes assessed using objective criteria? (5) If comparisons were being made, was there sufficient description of the groups? (6) Were the outcomes of people who withdrew described and included in the analysis? (7) Were outcomes measured in a reliable way? (8) Was appropriate statistical analysis used? **For qualitative studies:** (1) Was there congruency between the stated philosophical perspective between the research and the methodology? (2) Was there congruity between the research methodology and the research question or objectives? (3) Was there congruity between research methodology and data collection methods (4) Were there congruity between research methodology and representation and analysis of data (5) Were there congruity between research methodology and interpretation of results? (6) Was there a statement locating the researcher culturally or theoretically? (7) Was the influence of the researcher on the research and vice versa addressed? (8) Were participants and their voices adequately represented? (9)Was the research ethical according to current criteria or, for recent evidence of ethical approval by an appropriate body? (10) Did the conclusions drawn in the research report flow from the analysis or interpretation of the data? Each item was rated Y = Yes, N = No or U = Unclear. Unclear was awarded where not enough information was provided. High quality: meets ≥ 7 criteria, Moderate quality: meets ≥ 4 criteria, Low quality: meets < 4 criteria.
